# Diagnosis and phenotypic assessment of trimethylaminuria, and its treatment with riboflavin: ^1^H NMR spectroscopy and genetic testing

**DOI:** 10.1186/s13023-019-1174-6

**Published:** 2019-09-18

**Authors:** Nadia Bouchemal, Lisa Ouss, Anaïs Brassier, Valérie Barbier, Stéphanie Gobin, Laurence Hubert, Pascale de Lonlay, Laurence Le Moyec

**Affiliations:** 1 CSPBAT, UMR 7244, CNRS, Université Paris 13, Sorbonne Paris Cité, Bobigny, France; 2 Reference Centre for Metabolic Diseases, Necker-Enfants Malades Hospital, Imagine Institute, Université Paris-Descartes, APHP, Paris, France; 3 Service de Pédopsychiatrie, Necker-Enfants Malades Hospital, APHP, Paris, France; 4 Unité de Génétique moléculaire, Necker-Enfants Malades Hospital, APHP, Paris, France; 5 UBIAE, EA 7362, Univ Evry Université Paris-Saclay, Evry, France

**Keywords:** Trimethylaminuria, Olfactory reference syndrome, *FMO3* gene, Proton nuclear magnetic resonance spectroscopy

## Abstract

**Background:**

Trimethylaminuria (TMAU) is a metabolic disorder characterized by the excessive excretion of the malodorous compound trimethylamine (TMA). The diagnosis of TMAU is challenging because this disorder is situated at the boundary between biochemistry and psychiatry. Here, we used nuclear magnetic resonance spectroscopy to assess TMAU in 13 patients. We also sequenced the *FMO3* gene in 11 of these patients. Treatment with vitamin B2 was prescribed.

**Results:**

Two patients (aged 3 and 9 years at the initial consultation) had a particularly unpleasant body odor, as assessed by their parents and the attending physicians. The presence of high urine TMA levels confirmed the presence of a metabolic disorder. The two (unrelated) children carried compound heterozygous variants in the *FMO3* gene. In both cases, vitamin B2 administration decreased TMA excretion and reduced body odor. The 11 adults complained of an unpleasant body odor, but the physicians did not confirm this. In all adult patients, the urine TMA level was within the normal range reported for control (non-affected) subjects, although two of the patients displayed an abnormally high proportion of oxidized TMA. Seven of the 9 tested adult patients had a hypomorphic variant of the *FMO3* gene; the variant was found in the homozygous state, in the heterozygous state or combined with another hypomorphic variant. All 11 adults presented a particular psychological or psychiatric phenotype, with a subjective perception of unpleasant odor.

**Conclusions:**

The results present the clinical and biochemical data of patients complaining of unpleasant body odor. Contrary to adult patients, the two children exhibited all criteria of recessively inherited trimethylaminuria, suspected by parents in infancy. B2 vitamin treatment dramatically improved the unpleasant body odor and the ratio of TMA/Cr vs TMAO/Cr in the urine in the children. Other patients presented a particular psychological or psychiatric phenotype.

## Background

Trimethylaminuria (TMAU, also referred to as “fish odor syndrome” (FOS)) is characterized by an unpleasant body odor reminiscent of rotting fish. The condition is due to the excretion of abnormally high levels of the volatile tertiary aliphatic amine trimethylamine (TMA) in the urine, sweat and breath. TMAU is caused by mutations in the *FMO3* gene resulting in a reduction in TMA oxidation by the flavin-containing monooxygenase 3 enzyme [1]. TMA derives from either the bacterial metabolism of precursors (such as trimethylamine N-oxide (TMAO)) or the gut’s degradation of dietary choline, lecithin and (possibly) carnitine contained in sea fish, red meat, egg, beans and peas. Under normal dietary conditions, approximately 1 mg of TMA and 40 mg of TMAO per day are excreted in the urine [2]. TMAU is diagnosed by measuring the TMAO:TMA ratio in the urine. In normal subjects, 80% of the TMA is oxidized and then excreted mainly in the urine; in individuals with TMAU, less than 25% of the TMA is oxidized [2, 3]. The results may also be expressed as an “oxidizing ratio” (TMAO/(TMAO + TMA)), which is below 0.8 in affected individuals carrying two *FMO3*-inactivating mutations. Unaffected subjects on a normal diet should have an oxidizing ratio greater than 0.8 [4]. In a report by Chalmers et al. (2006) [5], TMAU was diagnosed in children on the basis of the TMA/creatinine (Cr), TMAO/Cr and TMA/TMAO ratios.

A variety of methods have been used to measure urine TMA and TMAO levels: proton nuclear magnetic resonance (^1^H NMR) spectroscopy [6], gas chromatography [7], electrospray ionization tandem mass spectrometry [8], direct infusion electrospray quadrupole time-of-flight mass spectrometry [9], and matrix-assisted laser desorption/ionization time-of-flight mass spectrometry [10]. High-resolution ^1^H-NMR spectroscopy provides a rapid evaluation of all H-containing substances in urine. One can therefore estimate TMA and TMAO levels simultaneously in a single experiment, unlike gas chromatography and mass spectrometry techniques [11]. As noted by Maschke et al. [12], the use of NMR avoids the need for sample pretreatments that might alter levels of volatile amine molecules.

It has been demonstrated that TMAU is associated with variants in the gene [13–18]. However, the degree of reduction in FMO3 enzyme activity appears to depend on the substrate studied [19–21]. The c.472G > A (p.Glu158Lys) variant has a high allele count in the general population (40%, depending on the geographic origin) [22], which was recently confirmed by whole-exome sequencing [23], and corresponds to a prevalence of approximately 10% for homozygous individuals. In some populations, this variant has been found in linkage disequilibrium with another variant (c.923A > G, (p.Glu308Gly)). Although common in the population, these variants have been associated with impairments in metabolism [24–26]. When these variants are present on the same allele, they exert a more pronounced effect on FMO3 function [19] and can even cause mild or transient forms of TMAU [15].

In some affected patients, TMAU is suspected at birth. However, the condition usually becomes apparent when the infant starts to eat foods with a high choline content (i.e., eggs, liver and other offal) or those containing TMAO (from marine fish), as the patients are unable to effectively reoxidize the TMA formed by the degradation of ingested TMAO. TMAU can also be accentuated by excessive sweating caused by intensive physical exercise, stress, or (in women) hormonal variations before and during menstruation [27].

Mitchell and Smith proposed a classification system for the various subtypes of TMAU [28]: (i) primary genetic TMAU (with autosomal recessive inheritance), (ii) acquired TMAU, which occurs in childhood (possibly after viral hepatitis), (iii) transient childhood TMAU, (iv) transient TMAU associated with menstruation, and (v) precursor overload TMAU (reported in a patient treated with betaine for homocystinuria, leading to an increase in TMA levels [29], and in another patient treated with L-carnitine [30]).

TMAU has a major psychosocial impact. In adulthood, affected individuals often face relationship problems and suffer from low self-esteem; in turn, this may lead to social isolation, alcoholism, depression and even suicidal tendencies. In childhood, affected individuals tend to be shunned, ridiculed, or bullied at school, leading to aggressive or disruptive behavior and poor educational performance. FOS should not be considered as a benign or “social” condition. It can affect infants, children [5, 6] and adults [31].

Differential diagnosis with regard to psychological disorders is crucial. Most people complaining of unpleasant body odor are ultimately diagnosed with olfactory reference syndrome (ORS) [32]; this is characterized by a preoccupation with body odor that results in significant distress and functional impairment. Olfactory reference syndrome appears to overlap with conditions such as schizophrenia, social phobia, obsessive compulsive disorder [33] and delusional disorder (sometimes with a single, delusional belief) [34]. The phenomenological overlap with anxiety and depression emphasizes the need to pay more attention to the differential diagnosis of TMAU [35]. Wise et al. found that African-American women are particularly likely to complain of idiopathic malodor [36]. In Japan, a condition similar to ORS has long been recognized as “taijinkyofusho”; this was believed to be a specific Japanese, culture-bound syndrome [37], although no reference to environmental factors (such as a fish-based diet) had been made. Olfactory reference syndrome is characterized by high morbidity and a search for nonpsychiatric treatment [38]. It has been reported that treatment with selective serotonin reuptake inhibitors and antipsychotic drugs reduces the symptoms of ORS [39].

People with TMAU can receive an appropriate dietary treatment by excluding TMA precursors. Alternatively, dietary supplementation with riboflavin (vitamin B2, a cofactor required for the activity of acyl-CoA dehydrogenases [40]) reduces TMA excretion and body odor in some patients with TMAU [29].

In the present study, we performed a clinical, ^1^H-NMR and genetic investigation of adults and children consulting our metabolic disease clinic for a complaint of unpleasant body odor. The two individuals with confirmed TMAU (two unrelated children) were treated with vitamin B2, and the treatment’s impact on urine TMAO and TMA levels was assessed.

## Results

### Patients

The biochemical and genetic characteristics of the study population are summarized in Table 1, and the clinical characteristics are presented in Table 2. The study population included 11 adults (7 females, 4 males; mean ± standard deviation (range) age: 34 ± 12.3 (20–62)) and two children (boys aged 3 and 9 at the initial consultation). The educational level was low in two adults, medium in seven, and high in one.

**Table 1:** Biochemical and genetic characteristics of the study population, including NMR ratios, the total number of abnormal NMR ratios (out of the four), and the number of abnormal NMR ratios after excluding the TMAO/Cr ratio (highly dependent on the diet factors)

Subjects	Mutations	Amino acid changes	TMA/TMAO *N* < 0.1	TMAO/TMA + TMAO *N* > 0.8	TMAO/Cr (mmol/mol) N 50–1000	TMA/Cr (mmol/mol) *N* < 10	Number of out-of-range NMR ratios (out of 4)	Number of out-of-range NMR ratios (out of 3, excluding TMAO/Cr)
Patient #1 (adult)	heterozygous c.472G > A	heterozygous p.Glu158Lys	0.13	0.88	11.16	1.46	2	1
Patient #2 (adult)	homozygous c.472G > A	homozygous p.Glu158Lys	ND	ND	ND	ND	ND	ND
Patient #3 (adult)	heterozygous c.472G > A heterozygous c.923A > G	heterozygous p.Glu158Lys heterozygous p.Glu308Gly	0.04	0.96	39.66	1.60	1	0
Patient #4 (adult)	ND	ND	0.37	0.73	7.00	2.56	3	2
Patient #5 (adult)	heterozygous c.472G > A heterozygous c.923A > G	heterozygous p.Glu158Lys heterozygous p.Glu308Gly	0.03	0.97	49.57	1.63	1	0
Patient #6 (adult)	homozygous c.472G > A	homozygous p.Glu158Lys	0.05	0.95	70.89	3.65	0	0
Patient #7 (adult)	no mutation	no mutation	0.15	0.87	119.27	17.84	2	2
Patient #8 (adult)	ND	ND	ND	ND	ND	ND	ND	ND
Patient #9 (adult)	homozygous c.472G > A	homozygous p.Glu158Lys	0.04	0.96	51.61	2.01	0	0
Patient #10 (adult)	no mutation	no mutation	0.10	0.91	32.13	3.11	1	0
Patient #11 (adult)	heterozygous c.472G > A	heterozygous p.Glu158Lys	0.08	0.93	3.94	0.30	1	0
Patient #12 (child)	heterozygous c.458C > T heterozygous c.419 T > C	heterozygous p.Pro153Leu heterozygous p.Phe140Ser	1.14	0.41	86.00	123.00	3	3
Patient # 13 (child)	heterozygous c.458C > T heterozygous c.769G > A	heterozygous p.Pro153Leu heterozygous p.Val257Met	1.15	0.54	25.50	29.30	4	3

N correspond to normal

*ND* not determined

**Table 2:** Clinical characteristics of the study population

Patient	Sex	Age	Educational level	Age at symptom onset	work/ academic problems	GAF score	Self-perception of odor	Odor directly perceived by a third party	Interpretation	Source of odor	Odor control strategies	Depressive symptoms	Impact on sex life
Patient #1	F	adult	medium	adulthood	no	65	yes	by a friend	yes	sweating	food eviction	no	yes
Patient #2	M	adult	high	adolescence	ND	55	no	no	yes	not known	showers	no	yes
Patient #3	M	adult	low	adulthood	no	65	no	by a friend	yes	not known	showers, food eviction	no	yes
Patient #4	F	adult	medium	childhood	yes	50	yes	by a friend	yes	skin, breath	food eviction, perfumes, antibiotics	yes	yes
Patient #5	F	adult	medium	adulthood	no	65	no	no	yes	not known	none	yes	yes
Patient #6	F	adult	medium	adolescence	no	60	no	no	yes	not known	showers	yes	yes
Patient #7	F	adult	low	adulthood	no	90	no	by a friend	no	genital	showers, food eviction	yes	no
Patient #8	M	adult	medium	adulthood	yes	30	no	no	yes	not known	none	no	yes
Patient #9	F	adult	medium	adulthood	no	50	no	no	yes	sweating, breath	perfumes, showers, food eviction	no	no
Patient #10	F	adult	medium	adulthood	no	60	no	no	yes	not known	none	yes	no
Patient #11	M	adult	not recorded	adulthood	no	60	yes	no	yes	sweating	food eviction	yes	yes
Patient #12	M	child	school age	< 3 years	no	90	no	by parents and clinicians	no	sweating, scalp, hands	none	no	not relevant
Patient #13	M	child	school age	< 3 years	no	90	no	by parents and clinicians	no	scalp, sweating	none	no	not relevant

*GAF* Global Assessment of Functioning. Interpretation: persecution ideas, interpretation

The clinicians, who specialized in inherited metabolic diseases, detected an unpleasant body odor in the two children but not in the 11 adult patients at the time of the visit. The first symptoms appeared after the age of 16 in all but one adult (at the age of 9 years) and during the first year of life in both children. Both children are suspected of having TMAU by their parents, who had noticed an unpleasant body odor soon after birth. The children were referred to our clinic at the ages of 3 and 9, respectively. The two children had been educated in a normal school setting and did not have any psychological problems. The main differences between the two pediatric cases and the 11 adult cases were as follows: the confirmation of an unpleasant body odor by the parents and the clinicians, the age at consultation, and the age at symptom onset. The levels of complaint and discomfort were high for the adults but low for the children and their families.

All adult patients provided a description of their symptoms. Malodor was reported directly by three adults. In ten cases, the adult patient believed that he/she had malodor because of remarks or behavior by friends interpreted as a sign of discomfort (e.g., “he is leaving the room because I have malodor”). Eight of the 11 adults used a variety of strategies to decrease the supposed odor. The source of the odor was reportedly sweat in 5 cases, the breath in 2 cases, the genitals in one case, the scalp in two cases (the children), the skin in one case (an adult) and the hands in one case (a child). Six patients could not name a source. The impact of the malodor was high, i.e. a GAF score below 70 in all but three cases (the two children and an adult who did not interpret the relatives’ behavior). Only 2 adult patients had work/academic problems. All adult patients had a history of psychiatric conditions, including depressive symptoms in 6 cases. Eight of the adults reported that their perceived malodor had an impact on their sex life.

### NMR spectra

**Fig. 1:**
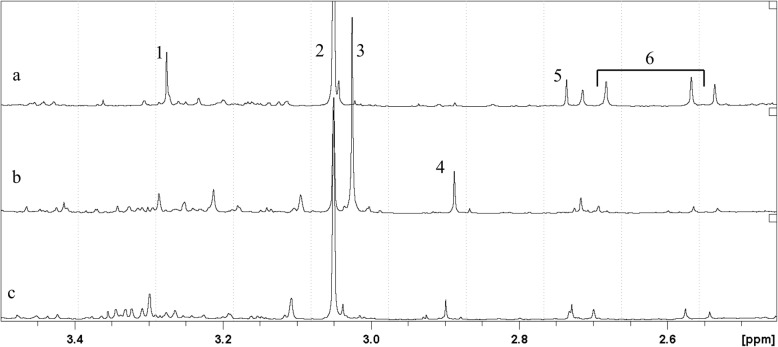
500 MHz ^1^H spectra of the urine of the two patients with FOS (**b** and **c**) and of a healthy subject (**a**). Assignments are as follows: 1 = trimethylamine-N-oxide (TMAO): 3.27 ppm; 2 = creatinine (Cr): 3.06 ppm; 3 = creatine (Cn): 3.04 ppm; 4 = trimethylamine (TMA): 2.92 ppm; 5 = dimethylamine (DMA): 2.73 ppm; 6 = citrate (cit): 2.56 and 2.72 ppm

Urine samples from all subjects attending our medical unit with suspected TMAU were analyzed using NMR. Figure 1 shows the ^1^H-NMR spectra of urine samples from three representative subjects: an adult in whom malodor was not detected by the clinicians (Fig. 1a) and the two children in whom malodor was detected by the parents (Fig. 1b and c). The TMA peak at 2.92 ppm was much more intense in the children’s urine samples than in the adult’s urine sample.

The dimethylglycinurie may be in a form of disease similar to FOS [41]. In our study, the dimethyglycine was detectable in urine spectra but the level of this metabolite was never found high enough to be correctly quantified.

For adult patients, the metabolite ratios are given in Table 3. The mean values were in line with the ranges previously reported for healthy adults, as confirmed by the calculated confidence intervals (Table 3). Individual patient data are reported in Table 1. The TMAO/Cr ratio was below 50 for 6 of the 11 adults, TMA/Cr was over 10 in 1 case, TMA/TMAO was over 0.1 in 3 cases, and TMAO/(TMA + TMAO) was below 0.8 for 1 case. Two patients had two out-of-range ratios, a third patient had three out-of-range ratios, and none had four out-of-range ratios. Given that the TMAO levels depend closely on the composition of the diet (e.g., a choline-rich diet), greater variability can be expected. Consequently, we counted the number of out-of-range ratios after excluding TMAO/Cr (Table 1); with this restriction, two patients (#4 and #7) showed two out-of-range ratios, and one patient (subject 1) had one out-of-range ratio value.

**Table 3:** Mean [95% confidence interval] metabolite ratios for the adult patients (#1 to #11)

Metabolite ratio	Mean	95% confidence interval	*P*-value	Reference value
TMAO/Cr (mmol/mol)	65.5	42–89	2.10^−6^	50–1000
TMA/Cr (mmol/mol)	7.8	1–14	3.10^−2^	< 10
TMA/TMAO (mol/mol)	0.13	0.06–0.19	7.10^−4^	< 0.1
TMAO/TMA + TMAO (mol/mol)	0.90	0.85–0.94	2.10^−16^	> 0.8

The normal ranges for healthy controls were TMAO/Cr ranging from 50 to 1000, TMA/Cr < 10, TMA/TMAO < 0.1 (reported by Chalmers et al. [5]) and TMAO/(TMA + TMAO) > 0.8 (reported by Eugène [4])

The results obtained for each sample collected from the 2 children are presented in Table 4. For patient #12, all the ratios were out of the normal range, with the exception of TMAO/Cr, which suggests the persistence of residual oxidizing activity. This finding was confirmed by the high urine TMAO/Cr ratio measured after an amine-rich-diet overload test. After vitamin B2 treatment, the ratios were within the normal ranges, with the exception of the TMAO/Cr ratio, which was abnormally low. For patient #13, two samples were available. The first sample (obtained before the vitamin B2 treatment had been initiated) yielded abnormal values for all ratios. After vitamin B2 treatment, all the values were improved. Similar results were observed 2 and 3 months later. In both pediatric cases, the body odor was normalized by treatment with 50 mg of vitamin B2 per day. The metabolic disorder specialist recommended vitamin B2 treatment for all adult patients, but only one initiated the treatment. In the latter case, vitamin B2 treatment had no impact on the perceived odor and thus was withdrawn.

**Table 4:** Metabolite ratios for the pediatric patients (#12 and #13) at different time points (with and without overload and with and without vitamin B2)

Metabolite ratio	Patient #12 Time 0 with dietary overload	Patient #12 3 months without dietary overload without vitamin B2	Patient #12 9 months with vitamin B2	Patient #13 Time 0 without dietary overload without vitamin B2	Patient #13 2 months with vitamin B2	Patient #13 3 months with vitamin B2
TMAO/Cr (mmol/mol)	1702	86	28	25.5	67.3	55.7
TMA/Cr (mmol/mol)	192	123	9	29.3	22.3	29
TMA/TMAO (mol/mol)	0.113	1.14	0.32	1.15	0.33	0.52
TMAO/(TMA + TMAO) (mol/mol)	0.90	0.411	0.76	0.54	0.75	0.66

The normal ranges for healthy controls were TMAO/Cr ranging from 50 to 1000, TMA/Cr < 10, TMA/TMAO < 0.1 (reported by Chalmers et al. [5]) and TMAO/(TMA + TMAO) > 0.8 (reported by Eugène [4])

### Genetic analysis

The *FMO3* gene variants and the patients’ TMA metabolite ratios are given in Table 1. *FMO3* gene sequencing revealed variants for 9 of the 11 tested patients (9 adults and 2 children).

The two unrelated children each carried compound heterozygous variants: a c.458C > T, (p.Pro153Leu) variant inherited from their respective fathers, and either the c.769G > A, (p.Val257Met) or c.419 T > C, (p.Phe140Ser) variant inherited from their respective mothers. An in silico analysis predicted that these variants are pathogenic. The c.458C > T variant (p.Pro153Leu) has previously been reported in individuals with inherited TMAU [13, 42, 43], and functional analysis demonstrated its impact on enzyme activity [14, 19, 44]. The c.769G > A (p.Val257Met) variant has also been reported, but it did not significantly affect FMO3 activity [45].

Seven of the 9 tested adults carried the variant c.472G > A (p.Glu158Lys). Three individuals were homozygous for this variant, and the other four were heterozygous. Two of the three homozygous patients were assessed with NMR, but neither displayed abnormal TMA metabolite ratios. Two of the heterozygous patients also carried another variant (c.923A > G (p.Glu308Gly)). We could not determine whether the two variants were *in cis* or *in trans*. Both variants have already been reported in individuals with TMAU as common polymorphic variants, and their role in TMAU was discussed [42]. Neither of the two patients displayed an abnormal oxidation profile. Two adult patients presented the most abnormal NMR ratios: one did not have a variant in the *FMO3* gene (patient #7), and the other was not investigated (patient #4, who refused).

## Discussion

Individuals with TMAU may sporadically produce malodor, despite good hygiene. The psychosocial impact of TMAU can be considerable. However, TMAU is difficult to diagnose because (i) detection of a metabolic problem requires specialized measurement techniques and (ii) metabolite production is diet-dependent and thus varies over time. In this study, the characteristics of TMAU in the children differed from those in the adults, as the children and adults differed in terms of the age at symptom onset, the objective detection of malodor by the clinicians, and the variants in the *FMO3* gene. The patients’ psychological profiles were also different. All the adults presented with a psychological history, and most reported an impact on their sex life. Two of the three variants found in the children had been reported previously [13, 46]. The third is considered to be pathogenic on the basis of a predictive in silico analysis and has not previously been reported in the general population. These variants might well be pathogenic mutations and were distinct from the common hypomorphic variants c.472G > A (p.Glu158Lys) and c.923A > G (p.Glu308Gly) detected in several of our adult patients. These latter variants have been extensively discussed in the literature as *FMO3* polymorphisms due to their high frequency in the general population and their moderate impact on enzyme activity [19, 21]. A diagnosis of TMAU in individuals carrying the latter variants is questionable in view of (i) the measured urine levels of TMA and (ii) reports that TMAU symptoms may be caused by a “spectrum” of changes to the gene, ranging from disease-causing variants to nonbenign polymorphisms associated with less severe symptoms [44, 47, 48]. Many adults suffer from odor complaints (e.g., in Shimizu et al.’s recent survey of 640 Japanese patients with self-reported TMAU [18]), although the malodor is usually not recognized by a physician [23]. However, the patients often do not display a normal urine TMAO/TMA ratio and thus meet the criteria for TMAU [23]. Moderate reductions in FMO3 catalytic activity (depending on the substrate) have been reported in an in vitro study using cDNA mutants [19]; the c.472G > A (p.Glu158Lys) variant was detected in patients with obvious psychiatric disease (e.g., the presence of isolated persecution ideas and interpretations). The c.472G > A (p.Glu158Lys) and c.923A > G (p.Glu308Gly) variants were found together and were not directly related to the TMAU phenotype [15]. Other studies have compared mutation frequencies in different ethnic groups [20, 21] and confirmed (in in vitro functional assays) that FMO3 N-oxidation activity may depend on the amine substrate in question (leading even to an increase in observed catalytic activity in some cases). Only 7 of the 640 volunteers with self-reported TMAU in Shimizu et al.’s study harbored variants in the *FMO3* gene, and only 19 of the 640 had less than 40% of the normal FMO3 metabolic capacity [18]. In another study of 102 Japanese patients, the 13 patients with the most severe TMAU phenotype (assessed by the measurement of urine TMA and TMAO levels with gas chromatography) displayed 11 different genotypes for the *FMO3* gene. More generally, the diagnosis of FOS is subject to much debate.

The ratios measured in our adult patients are in line with the values for healthy controls reported by Chalmers et al. [5] and by Eugène [4]. When considering our adult patients with out-of-range values, only two showed abnormal ratios (when excluding the TMAO/Cr ratio) and did not have physician-confirmed malodor. In one particular case (patient 4), the TMA/Cr ratio was in the normal range, but the TMAO/Cr ratio was low, which consequently lowered the TMAO/(TMA + TMAO) ratio and increased the TMA/TMAO ratio. This patient was the only one with symptom onset in childhood. Unfortunately, we have no genetic assessment for this patient. Consequently, we conclude that all ratios assessed in the present study are needed to confirm a diagnosis of TMAU and interpret possible alterations in TMA metabolism. The adult patients investigated in this study were very much like those described by Guo et al. (2017) [23]: Guo et al.’s 10 patients complained about a fish odor that could not be detected by trained sensory judges, and the *FMO3* variants detected in 7 of the 10 patients were homozygous or heterozygous and were only weakly correlated with the TMAO/TMA ratios measured in urine.

For the two children with physician-confirmed malodor, the samples obtained without amine overload and without vitamin B2 treatment yielded markedly out-of-range TMA/TMAO and TMAO/(TMA + TMAO) ratios; these abnormal values were essentially due to an elevated TMA/Cr value. A concomitant decrease in the TMAO/Cr ratio enhanced the variation in the other ratios; this is why Eugène proposed a diagnosis of TMAU based on the TMAO/(TMA + TMAO) ratio [4].

In patient #12, the overload diet induced an increase in the TMA/Cr ratio and a concomitant increase in the TMAO/Cr ratio. The relative increase in the level of the oxidized metabolite suggests that residual oxidizing activity is present in this patient. The marked effect of vitamin B2 in both children was evidenced by the TMA/Cr ratio, which fell drastically within a few weeks. Consequently, the TMAO/(TMA + TMAO) ratio rose to above the upper limit of the normal range, and the TMA/TMAO ratio fell. Following the normalization of the metabolite profile, the vitamin B2 treatment led to a drastic reduction in the malodor. In contrast, vitamin B2 treatment had no effect on the only adult who agreed to initiate it. The TMA/Cr ratio did not change significantly, whereas the TMAO/Cr ratio rose. A previous study of a betaine-treated patient with homocystinuria and malodor demonstrated the benefits of vitamin B2 treatment on TMAU [29]. The researchers showed that vitamin B2 administration was associated with reduced TMA excretion in some cases; this is presumably due to increased FMO3 activity when riboflavin acts as a cofactor. It has previously been demonstrated that NMR spectroscopy is a reliable means of detecting TMAU. The technique is rapid and requires only a small volume of urine (less than 1 mL). No sample preprocessing is required, which therefore maintains the sample’s biological composition. The limitation of this technique relates to the low availability of high-field NMR spectrometers, most of which are located in research units rather than in clinical biochemistry units.

However, given the large number of reported *FMO3* variants (which may or may not be associated with TMAU), we consider that the assessment of TMA and TMAO is essential for discriminating between a true *FMO3* enzyme deficiency with FOS during childhood on one hand and a complaint in adulthood in the absence of malodor confirmed by a third party. Importantly, other genes may be involved in or interact with TMAU; further studies are therefore needed to identify TMAU-causing variants in *FMO3* and/or in as-yet uncharacterized interacting genes [23].

## Conclusion

The present results showed that the amine content of frozen/thawed urine samples can be accurately measured using proton NMR spectroscopy. Considering the large variability of amine eliminated in urine, several ratios have to be calculated. Only two children met all criteria for TMAU, which had been suspected in infancy by the parents. Vitamin B2 treatment drastically reduced the malodor and normalized the TMA/TMAO ratio in urine. All the other (adult) patients presented with a clinical complaint arising in late childhood or in adulthood and not perceived by the physicians at the time of the visit. Their common polymorphic variants, not found in all, are unlikely to be functionally relevant and could mislead the diagnosis of TMAU. The biochemical validation of TMAU diagnosis can be made only for cases where (i) the odor is confirmed by the physician(s) or the parents, and (ii) the malodor begins during childhood. All cases suggestive of ORS (linked to a variety of psychiatric conditions [33, 34]) should be referred for psychological or psychiatric care so that the adults concerned can receive specific interventions for the condition’s psychic and social impacts.

## Materials and methods

### Patients

We performed a retrospective analysis of the medical records of all patients identified at Necker Hospital (Paris, France) with a diagnosis of TMA.

The 13 patients included in the present study had been referred to the metabolic disease unit at Necker Children’s Hospital (Paris, France). All complained of an unpleasant body odor noticed by themselves or their family and friends. All patients were interviewed by one of two physicians involved in inherited metabolic diseases. Patients were also systematically seen by the psychologist of the metabolic unit. They were referred to a psychiatrist, if appropriate. The study was approved by the local ethics committee at Necker Children’s Hospital. Clinical samples were registered with the Clinical Research Department (*Département de la Recherche Clinique et du Développement*) at the Paris Public Hospital Group (Assistance Publique, Hôpitaux de Paris) after the provision of written informed consent.

### Urine sample preparation

Urine samples were collected, immediately frozen at − 20 °C, sent to the NMR facility, and thawed at room temperature immediately before analysis. Six hundred microliter aliquots were used directly for ^1^H-NMR analysis in 5-mm diameter NMR tubes, together with 100 μL of deuterium oxide 99.96% (Eurisotop) as an internal field-frequency lock. Dietary overload (a 3-day amine-rich diet) was performed by patient #12. Eggs, cabbage, fish and crustaceans were recommended. The urine sample was collected on the fourth day.

### ^1^H NMR spectroscopy

NMR experiments were conducted in a Bruker AVANCE III spectrometer (Bruker Biospin) operating at 500 MHz with a 5 mm gradient indirect detection probe and a probe temperature of 300 K. The one-dimensional proton spectra were acquired with 64 scans, 32 K data points, and a spectral width of 5000 Hz. A conventional proton 90° pulse with a relaxation delay of 2 s was used. The water signal was suppressed by irradiating at the water resonance frequency (i.e., with a presaturation sequence).

Resonances were assigned by reference to a spectral database of standard chemical shifts [49]. The Cr resonance at 3.05 ppm was used as an internal chemical shift reference. Under these conditions, TMA resonance was detected at 2.92 ± 0.02 ppm, and TMAO resonance was detected at approximately 3.27 ± 0.03 ppm, depending on the urine’s pH.

The metabolite peaks for Cr, TMA and TMAO were quantified by integration. The total amounts of TMA and TMAO excreted were normalized against the amount of Cr eliminated to estimate the amount with regard to glomerular filtration. Next, the results were expressed as the following ratios: TMAO/Cr (mmol/mol), TMA/Cr (mmol/mol), TMA/TMAO (mol/mol) [5] and TMAO/(TMA + TMAO) (mol/mol) [4].

The normal values reported by Chalmers et al. [5] for healthy controls were TMA/Cr < 10, TMAO/Cr ranging from 50 to 1000, and TMA/TMAO < 0.1. The normal value reported by Eugène [4] was TMAO/(TMA + TMAO) > 0.8.

### *FM03* gene sequencing

The *FMO3* gene was sequenced for 11 of the 13 patients (9 adults and 2 children). Genomic DNA was extracted from leukocytes. The *FMO3* gene’s coding exons and intron-exon boundaries (NM_001002294.2) were amplified by standard PCR and analyzed by direct sequencing on an ABI 3100 automatic sequencer (Applied Biosystems, France). The primers used for PCR and sequencing were designed with Primer 3 software (http://frodo.wi.mit.edu/primer3/). The potential impact of variants at the protein level was predicted in silico using Alamut and Polyphen 2 (http://genetics.bwh.harvard.edu/pph2/) databases.

### Clinical assessments

Each patient’s medical history was assessed in a nonstructured interview. We assessed the severity of symptoms with the Global Assessment of Functioning (GAF) score (assessed according to the “American Psychiatric Association, (2000), Diagnostic and Statistical Manual of Mental Disorders, 4th ed, Text Rev. Washington-DC”), which is used by mental health clinicians to subjectively rate an individual’s social, occupational and psychological functioning. This numeric scale ranges from 100 (no impairment) to 1 (very severe impairment).

### Statistics

Mean ± SD values were calculated for the adult subjects. The 95% confidence interval was estimated using the single-sample t-test in the R commander package in R (www.r-project.org*)*. The threshold for statistical significance was set to *p* < 0.05.

## Data Availability

All data are available in the paper.

## Abbreviations

Cr: Creatinine
FMO3: Flavin-containing monooxygenase 3
NMR: Nuclear magnetic resonance
ORS: Olfactory reference syndrome
TMA: Trimethylamine
TMAO: Trimethylamine-N-oxide
TMAU: Trimethylaminuria
